# Clivus-Cervical Stabilization through Transoral Approach in Patients with Craniocervical Tumor: Three Cases and Surgical Technical Note

**DOI:** 10.3390/brainsci14030254

**Published:** 2024-03-05

**Authors:** Gervith Reyes-Soto, Alfonso Corona De la Torre, Kaori Guadalupe Honda Partida, Renat Nurmukhametov, Manuel De Jesus Encarnacion Ramirez, Nicola Montemurro

**Affiliations:** 1 Department of Head and Neck, Unidad de Neurociencias, Instituto Nacional de Cancerología, Mexico City 14080, Mexico; 2 Neurological Surgery, Peoples Friendship University of Russia, 103274 Moscow, Russia; 3 Department of Neurosurgery, Azienda Ospedaliero Universitaria Pisana (AOUP), University of Pisa, 56100 Pisa, Italy

**Keywords:** clivus, cervical spine, stabilization, cementation, metastasis, spine, neurosurgery

## Abstract

Craniocervical tumors lead to cervical pain, instability, and neurological symptoms, reducing the quality of life. Effective surgical intervention at the craniocervical junction (CCJ) is critical and complex, involving comprehensive approaches and advanced reconstructive techniques. This study, conducted at Mexico City’s National Institute of Cancerology, focused on three surgical cases that occurred in 2023 involving tumors at the CCJ: two chordomas and one prostate adenocarcinoma. We utilized a specialized technique: clivus-cervical stabilization reinforced with a polymethylmethacrylate (PMMA)-filled cervical mesh. Postoperatively, patients showed marked neurological recovery and reduced cervical pain, with enhanced Karnofsky and Eastern Cooperative Oncology Group (ECOG) scores indicating improved life quality. The surgical technique provided excellent exposure and effective tumor resection, utilizing PMMA-filled cervical mesh for stability. Tumoral lesions at the CCJ causing instability can be surgically treated through a transoral approach. This type of approach should be performed with precise indications to avoid complications associated with the procedure.

## 1. Introduction

Tumors of the craniocervical junction (CCJ) are defined according to the involvement of the condyles and/or the atlantoaxial spine. The metastatic disease affecting the craniovertebral junction only represents 0.5% of all spinal metastases, although the spine is the most frequent site of bone metastases, particularly in men between 40 and 65 years old [1]. Whereas, the incidence of primary tumors, such as chordomas, ranges from 0.18 to 0.84 per million persons per year and varies per country [2]. The management of these pathologies is complex, involving multimodal radiological studies and various surgical approaches [3,4]. The diagnosis of craniocervical tumors has been facilitated by modern neurodiagnostic imaging techniques, including flexion/extension radiography of the craniocervical region, magnetic resonance imaging (MRI) with or without contrast, magnetic resonance angiography (MRA), magnetic resonance venography (MRV), computed tomography (CT), and three-dimensional CT angiography (CTA) [5].

The quality of life of these patients is often significantly compromised, especially as the bone remodeling of the tumor itself causes bone pain, fractures, instability, focal neurological deficit due to spinal cord compression, and symptoms associated with systemic disease [6]. There are multiple options available in the surgical arsenal to treat lesions located at the CCJ. The selection of the optimal surgical strategy is fundamental to the operation’s success, as it maximizes the chances of achieving the surgical objectives and minimizes morbidity related to the surgery [5].

The transoral approach at the CCJ is traditionally used to treat rheumatoid pannus, some tumors with spinal cord compression, and basilar invagination [7,8,9]. It was first described in 1917 by Kanavel, who used it to remove a projectile at the CCJ. Subsequently, the microsurgical technique was popularized and refined by expert neurosurgeons such as Crockard and Menezes [10]. Studies recommend considering transmandibular approaches for primary malignancies of the anterior column in the context of reaching or crossing the C2–C3 disc plane [11]. Within the context of tumoral pathology, there are multiple oncological etiologies at the craniocervical level. Historically, chordomas were presumed to be more prevalent in the sacrum than at the skull base; however, evidence suggests an almost equal distribution at the skull base (32%), mobile spine (32.8%), and sacrum (29.2%) [12].

Currently, total surgical resection and high-dose adjuvant radiotherapy are the treatment options for chordoma [13]. Studies show that total and subtotal resection have better survival in patients with skull base chordomas compared to partial resection. However, performing a maximal surgical resection requires the surgeon to be familiar with the anatomy of the skull base and, more importantly, recognize that it is a bone-invading tumor and that intraoperative resection of the diseased bone is maximized while preserving some functions and minimizing residual lesions that may cause recurrence [14,15,16].

Patients with significant instability generally require combined approaches (a 360° fixation). It is well-accepted that odontoidectomy will result in instability of the spine (acute or late), requiring posterior fixation [7,12,13]. Publications on strategies for reconstruction and stabilization of the cervical spine include the use of autografts or allografts from the fibula, Harms cages, custom wire cages with anterior cervical plates, 3D printed cages, and placement of expandable cage at the CCJ [17,18,19,20]. Metastatic involvement of the CCJ and/or kyphosis and collapse affecting any region of the cervical spine are key factors influencing the decision to stabilize the spine, with a preference for posterior techniques in CCJ involvement and anterior techniques generally recommended in the subaxial cervical spine [21,22].

There are few reports on the use of clivus-cervical fixation in metastatic and primary tumoral disease of the CCJ, as well as no reports on the use of a mesh with cementation in this type of surgery. In this study, we present our surgical experience in a series of 3 craniocervical tumor cases, showing step-by-step this surgical technique.

## 2. Design/Methods

This study presents a series of 3 cases treated surgically in 2023 at the National Institute of Cancerology Oncological Spine Neurosurgery Module in Mexico City. Our analysis encompassed three cases: two with a histological diagnosis of chordoma and one of prostate adenocarcinoma. The primary goal of the surgical interventions was to stabilize the CCJ and relieve pressure on the compromised neurological structures. We employed a specialized surgical technique involving clivus-cervical stabilization, supported by the insertion of a cervical mesh filled with polymethylmethacrylate (PMMA). The intricacies of this surgical procedure are thoroughly described.

### Surgical Technique

**Figure 1:**
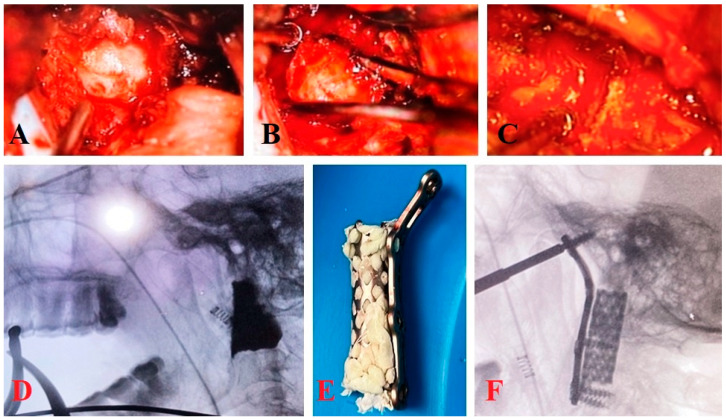
Transoral approach under microscope magnification (**A**–**C**) shows the resection of the odontoid and the anterior arch of C1. (**D**) Lateral fluoroscopic view following odontoidectomy. (**E**) Pre-modeled cervical mesh with 5 mm PMMA spheres. Anterior cervical plate screws are placed within the mesh with PMMA. (**F**) Lateral fluoroscopic view shows the placement of mesh on the clivus.

General anesthesia with endonasal intubation was administered and the patient was positioned in a supine position with the support of a Mayfield headrest. Prior to asepsis, three linked gauzes were inserted to prevent fluid passage into the airway, which was removed at the end of the surgical procedure. Oral asepsis was performed with Betadine. Prophylactic antibiotic administration was indicated orally for 7 days with Levofloxacin. A Dingman retractor was placed. With the aid of a microscope, a longitudinal incision of 3 to 5 cm was made along the midline from the soft palate to the hard palate, fixing the layers with sutures to the Dingman retractor to maintain exposure. This was followed by an incision of the posterior wall of the pharynx, after locating the arch of C1 and the vertebral body of C2. A mucosal and muscular plane was exposed through the pharyngeal mucosa, the constrictor muscles of the pharynx, and the longus collis and longus capitis muscles, laterally referring to the plane made with the support of reference sutures. A subperiosteal dissection was performed, exposing the tubercle of C1. A 15 mm section was resected from the midline of the anterior arch using a burr or Kerrison, providing access to the odontoid process (Figure 1A–C). Odontoidectomy was performed and verified by volumetry with iopamidol 300 and intraoperative fluoroscopy (Figure 1D). Subsequently, the previously measured mesh was cut and anatomically molded using CT images, ensuring the mesh corresponded with the anatomical area. The diameter corresponding to the cephalic end of the mesh was molded, given the smaller diameter of the clivus compared to the vertebral body of C3, thus giving the cervical mesh a somewhat conical shape.

The mesh was filled with PMMA using 5 mm diameter spheres. Extracorporeally, the anterior FULLE TM cervical plate was attached to the mesh structure using a screw. The team waited for the exothermic reaction of the PMMA to complete and then removed the screw and cervical plate from the mesh. This left the screw placement site empty so that it could later be placed in the patient (Figure 1E). The correspondence of the anterior cervical plate from the clivus to C3 was verified. Screws were placed in the clivus, previously measured at 4.5 × 12 mm, and in the C3 body at 4.5 × 16 mm, through the transoral approach with the support of an intensifier (Figure 1F).

**Figure 2:**
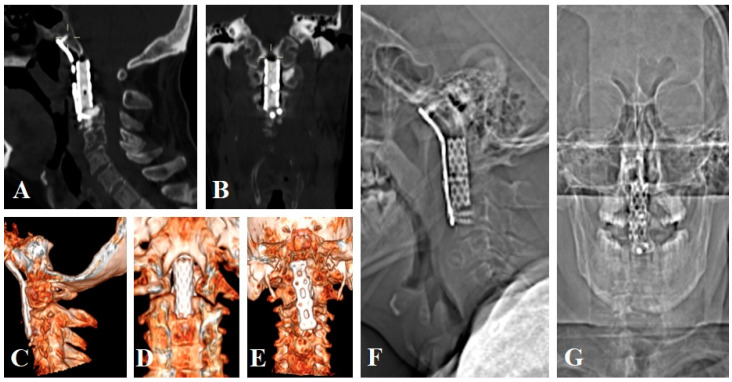
Postoperative CT scans (**A**,**B**), 3D reconstructions (**C**–**E**), and lateral (**F**) and anterior (**G**) x-rays show the clivus-cervical C3 stabilization and vertebrocementoplasty of the left lateral mass of C1 and the body of C3.

In all three cases, a transoral approach and clivus-cervical stabilization were performed as the primary surgical method. Only in one case was it decided to perform a posterior cervical approach and an occipitocervical stabilization plus laminectomy at the same surgical time. In all cases, it was decided to perform a post-surgical control CT scan the following day to verify the proper placement of the surgical material (Figure 2).

During surgery, we prefer the oral intubation way, because we think that nasal intubation may obstruct the clivus access. The closure technique after the transoral approach was performed with sutures in two layers (muscular and mucosal layer) with a 3-0 long curved monocryl needle; the uvula was divided by the paramedial line and the same reconstructive way. All patients begin oral intake with cold liquids 24 h after surgery and with pureed foods 3 to 7 days after surgery. All patients used a Philadelphia collar before and after surgery and until the time of radiotherapy.

## 3. Results

### 3.1. Clinical Cases

#### 3.1.1. Clinical Case 1

**Figure 3:**
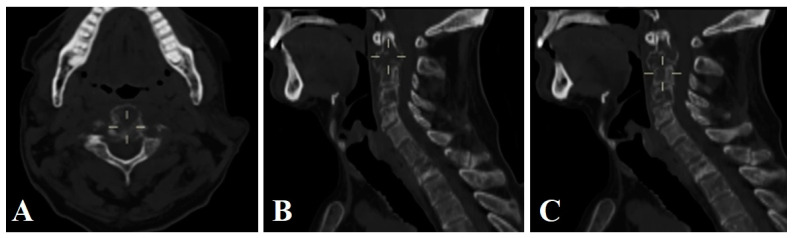
Axial (**A**) and sagittal (**B**,**C**) CT scans lytic lesions of cervical spine from C1 to C4, affecting the whole body of C2 and the odontoid process.

A 59-year-old male patient with a history of prostatic adenocarcinoma was admitted to our institution for cervicalgia with a VAS (Visual Analog Scale for Pain) of 8/10, even with the use of opioids, without integrating a spinal cord syndrome; he was comprehensively evaluated by different services, finding pulmonary and retroperitoneal metastases in a thoracoabdominal tomography, starting treatment with steroids. After performing MRI and CT scans of the spine, a pathological fracture of C2 was diagnosed, with lytic images predominantly in the lateral mass of C1, vertebral bodies of C3 and C4, and thoracic vertebral bodies (Figure 3).

First-line chemotherapy with Degarelix was performed. Subsequently, the patient began to experience neurological deterioration, presenting with weakness in the upper and lower limbs, C4–C7 3/5 and paresthesias from C3–C7, and L2-S1 3/5, with hyperreflexic bicipital, tricipital, and patellar reflexes, control of sphincters, functional scale scores of Karnofsky 50%, Eastern Cooperative Oncology Group (ECOG) 3, Tokuhashi score of 8 points and SINS 12 points. According to the calculator of the Global Spine Tumour Study Group (GSTG), the estimated percentage of post-surgical life survival for the patient was 23% at three months, 41% at six months, 61% at 12 months, and 78% at 24 months. Given the conditions of the neurological deficit, it was decided to start surgical treatment according to the Neurologic, Oncologic, Mechanical Instability, and Systemic Disease (NOMS) system. The decision was made to perform an initial intervention through a transoral approach and clivus-cervical stabilization plus vertebrocementoplasty of C3 and the lateral mass of C1, leaving the possibility of posterior cervical stabilization for a possible second surgical phase.

#### 3.1.2. Clinical Case 2

**Figure 4:**
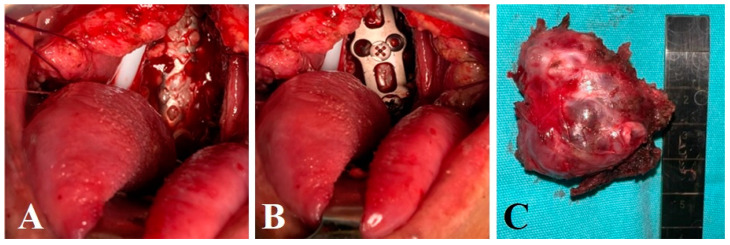
Transoral approach shows pre-modeled cervical mesh (**A**) and anterior cervical plate (**B**) placed with screws. Axial (**C**) The surgical specimen measures 5 × 6 cm, approximately 60 cc in volume, and weighs 16 g with a firm consistency.

A 68-year-old male patient, with a diagnosis of chordoma and posterior occipitocervical instrumentation without decompression 3 years before, was admitted to our institution as he presented cervicalgia and paresthesias in bilateral thoracic limbs. An MRI was performed, showing a ventral tumor of C1, C2 measuring 5 × 7 cm causing spinal cord compression. Clinically, the patient exhibited asymmetric muscle strength along the path of C5 and C6 3/5, hypoesthesia present in the dermatomes of C4, C5, and C7 bilaterally, with no signs of myelopathy, sphincter control, functional scale scores of Karnofsky 90%, ECOG 1, Tokuhashi score of 12 points and SINS 14 points. Surgical treatment with tumor excision and clivus-cervical stabilization was proposed to the patient, using the technique described above. A white-pearly lytic lesion with characteristics of a chordoma was found in the prevertebral plane. Excision was carried out while preserving the capsule (Figure 4), with the histological diagnosis confirming the presence of a metachronous chordoma. Since the patient was already previously fitted with occipito-cervical stabilization, he was referred to the radiotherapy unit to continue with the established oncological treatment.

#### 3.1.3. Clinical Case 3

**Figure 5:**
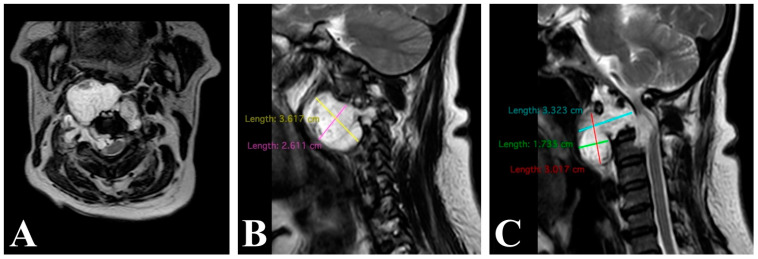
Axial (**A**) and sagittal (**B**,**C**) gadolinium-enhanced T1-weighted cervical spine MRI show C1–C2 lesion with more than 80% of spinal cord compression.

A 68-year-old female patient with no prior oncological diagnosis was admitted to our institution following cervicalgia for 4 years, with no improvement with drugs, and exacerbation in the last 10 months. She progressed with neurological deterioration and paresthesias in thoracic and pelvic limbs and loss of ambulation occurred 2 months ago. Following an MRI, a diagnosis was made of a CCJ tumor causing spinal compression, which, based on its imaging characteristics, is presumed to be a C2 chordoma (Figure 5).

The physical examination of the patient showed a bilateral C5-T1 3/5 muscle strength with myelopathy data, sphincter control, and functional scale scores: KARNOFSKY 40%, ECOG 4, SINS score of 15 points. Given the high risk of increased spinal cord compression, surgical treatment with tumor excision and clivus-cervical stabilization was proposed to the patient. Craniocervical instrumentation plus laminectomy of C1, C2, and C3 and clivus-cervical stabilization were performed using the surgical technique described above. A white-pearly lesion with characteristics of a chordoma was found in the prevertebral plane and the tumor histology was confirmed with pathology.

Table 1 shows full details of all three cases.

### 3.2. Outcome and Surgical Complications

The three patients, two diagnosed with chordoma and one with prostate adenocarcinoma, underwent successful clivus-cervical stabilization. Postoperatively, all patients exhibited marked improvements in their neurological status. Symptoms of cervical pain, which had been a significant preoperative complaint, were considerably alleviated.

The average hospital stay post-surgery was four days, reflecting a relatively quick recovery period for a procedure of this complexity. Oral intake was well-tolerated by the second postoperative day. No postoperative complications of wound dehiscence or exposure of the surgical material were reported, indicating successful wound healing and surgical technique. Throughout the short to medium-term follow-up period (6 months), no major surgical complications were reported. The patients tolerated the procedure well, with minimal postoperative discomfort. Furthermore, there were no reports of neurovascular complications, which are often a concern with surgeries in such complex anatomical regions. Though limited by the follow-up duration, long-term outcomes suggest sustained symptomatic relief and spinal stability. However, further studies are warranted to fully assess the long-term efficacy and potential late complications of this surgical approach.

### 3.3. Surgical Technique and Radiological Follow-Up

The transoral approach allowed for excellent exposure to the surgical field, extending from the clivus to the C4 vertebral body. This exposure was pivotal in enabling comprehensive tumor resection while maintaining the integrity of vital neurovascular structures. The use of a PMMA-filled cervical mesh in the stabilization procedure provided robust structural support and stability, crucial for the patient’s postoperative recovery and spinal integrity. Postoperative CT scans played a vital role in confirming the correct placement of the surgical materials and the success of the stabilization. These scans revealed a satisfactory alignment and stabilization of the CCJ, correlating well with the clinical improvements observed in the patients. In addition, we encourage the use of dynamic X-ray imaging approximately 3 months after surgery, excluding the use of a Philadelphia collar, to validate fusion success, along with CT scans.

## 4. Discussion

This study’s primary objective was to assess the efficacy and safety of clivus-cervical stabilization through a transoral approach in patients with oncologic pathology at the CCJ. The rarity of such cases necessitates a nuanced understanding of both the surgical technique and the pathophysiology of the involved neoplasms.

Within the literature, there are few reports of clivus-cervical stabilization with cervical plate and mesh with cementation in oncological pathology [23,24,25,26,27,28,29]. To our knowledge, the reported cases for treatment are expanding implants or meshes with cementation in tumors and metastases of the subaxial cervical spine. Regarding the presence of metastases in the CCJ, there is not enough information on surgical treatment since they cause instability and spinal cord compression [23,24,25].

Goel and Karapurkar [26] presented a case report of basilar invagination, where treatment with transoral odontoidectomy was performed, with fixation of a plate and screws from the clivus to the cervical body C3 [27]. Similarly, Ahsan and colleagues [28] presented a case of a C2/3 chordoma where a Trotter approach was performed, allowing for broad exposure, given the Le Fort I type mandibular osteotomy that must be performed in this type of approach. However, the authors mark as a limitation the poor lateral exposure obtained, which did not allow them to perform the total resection of the tumor [28].

Wewel [29] presented a case report of a C2 chordoma in which treatment was carried out with a total anteroposterior spondylectomy of C2 through a transoral anterior and posterior occipito-cervical instrumentation approach, using an expandable box filled with crushed allograft with integrated fixation from the clivus to C3. In our cases, the execution of a transoral approach allowed adequate exposure of the surgical field from the clivus to the vertebral body of C4, similarly allowing lateral reach for adequate tumor resection. So far, we have not presented complications such as wound dehiscence or short and medium-term exposure of material. However, these surgical procedures are characterized by high morbidity: cerebrospinal fluid (CSF) leakages (10–12%); meningitis (3–5%), ventriculitis (0.8–1%), injury of the spinal cord (1–3%), hemorrhage, dysphagia (3–5%), dyspnea, wound infection, instrumentation failure (4–7%), injury of the vertebral artery (0.2–0.5%) [30,31,32,33,34,35]. Chordomas are locally destructive and have high recurrence rates. The current standard of care for skull base chordomas is safe maximal resection, with an emphasis on preserving function, followed by radiotherapy [36,37]. Most of the related studies are case reports and there is little published information on metastasis to the high cervical spine and its ideal treatment. Regarding our results, patients had an immediate improvement in neurological status and cervical pain, oral intake was tolerated on the second day, and they were discharged on the fourth day with good wound evolution. Radiotherapy is used not only as adjuvant therapy after surgery but also as a treatment of choice for patients with inoperable tumors [38,39,40,41]. The treatment must be individualized taking into account the volume of the tumor, its localization, and prior use of radiation. Despite this, Napieralska and Blamek [42] did not observe a clear correlation between the dose delivered and the treatment effect. Other researchers found a correlation between the applied total dose and OS and local control [39], whereas Koga et al. [43] reported a statistically significant difference between patients treated with Gamma Knife with higher and lower doses (18 Gy vs. 12 Gy).

The use of PMMA-filled cervical mesh in our study has shown promising results in terms of structural support and stability. This aligns with advancements in spinal surgery, where the use of innovative materials has enhanced the outcomes of complex procedures [44,45]. The choice of PMMA over other materials such as hydroxyapatite (HA) or bone grafts is influenced by several factors. Although HA is known for its bioactive and osteoconductive properties that support bone growth, it may not provide the immediate structural stability required in some cases. Likewise, autologous bone grafts, considered the gold standard for promoting bone fusion due to their osteogenic, osteoinductive, and osteoconductive properties, have limitations such as donor site morbidity and limited availability. PMMA is chosen for its ability to provide immediate structural stability, ease of application, and the ability to precisely fill irregular defects without the risk of resorption. This makes PMMA particularly useful in surgeries where rapid stabilization of the spine is needed, such as in tumor resection surgeries.

The detailed description of our surgical technique is aimed at assisting surgeons in navigating similar complex cases, emphasizing the importance of preoperative planning and intraoperative precision. For metastatic or recurrent tumors, the purpose of the surgery is often palliative, and adjuvant radiotherapy and chemotherapy are administered after surgery. For primary bone tumors of the spinal column, surgery generally requires that the tumor be completely removed to achieve the purpose of cure or long-term tumor-free survival [46,47].

### 4.1. Incorporating Advanced Surgical Technologies

The success of complex surgical interventions, such as clivus-cervical stabilization through a transoral approach, is significantly enhanced by using advanced technologies. In our study, surgical microscopes played a pivotal role in achieving precision and safety [48]. The surgical microscope has been a mainstay in spinal surgery, providing enhanced magnification and illumination, which are crucial in navigating the intricate anatomy of the CCJ [49]. The microscope’s depth of field and clarity facilitate the meticulous dissection and manipulation required in such delicate areas. However, introducing the exoscope into spinal surgery represents a paradigm shift [50]. As a high-definition, 3D visualization system, the exoscope offers several advantages over the traditional microscope [51]. Its ergonomic design allows for a more comfortable surgeon posture, potentially reducing fatigue in lengthy procedures. Additionally, the exoscope’s ability to project the surgical field onto a screen enables the entire surgical team to view the operation, fostering a more collaborative environment [52].

Utilizing these technologies in our case series contributed to the precision of the surgical technique, particularly in achieving adequate tumor resection and in the placement of PMMA-filled cervical mesh. The enhanced visualization aids in minimizing the risk of injury to adjacent neurovascular structures, a critical consideration in transoral surgeries [50]. Therefore, improving the rates of total and near-total resection may be a key factor in achieving high rates of local control and survival in the current study. The use of augmented reality and telemedicine can improve the surgical outcome of these patients [53,54]. In conclusion, maximal surgical resection is the most important independent prognostic factor for manageable skull base chordomas.

### 4.2. Limitations of the Study

This study has several limitations. First, this study reflects the experience of a single institution. The study is based on a limited number of cases (three patients), which may not provide a comprehensive representation of the broader patient population with similar conditions. This small sample size limits the generalizability of the findings and may not capture the full spectrum of potential outcomes and complications associated with the surgical technique. Second, the absence of a control group makes it difficult to draw comparative conclusions about the efficacy of the clivus-cervical stabilization through a transoral approach versus other surgical methods. Third, the follow-up period in this study is not specified, but longer-term follow-up is essential to assess the durability of the surgical outcomes, the long-term complications, and the recurrence rate of the tumors. In addition, this study includes different types of tumors (chordomas and prostate adenocarcinoma), which may have different biological behaviors and responses to surgery. The study highlights the use of advanced technologies such as microscopes and exoscopes, which may not be readily available in all surgical settings, particularly in low-resource environments. This limits the applicability of the technique in such settings. Multi-center studies are needed to validate these findings across different settings.

## 5. Conclusions

Tumoral lesions at the CCJ causing pain, neurological deficits, and instability can be surgically treated through a transoral approach. This type of approach should be performed with precise indications to avoid complications associated with the procedure. Adequate results can be obtained through this route, with very good exposure to prevertebral tumoral lesions, thus avoiding complications related to the neurovascular structures present.

## Figures and Tables

**Table 1:** Reported cases of transoral tumoral resection and cervical-clivus fixation.

No.	Age/ Sex	Comorbidities	Tumor Localization	Histology	SINS (pts)	Preop. Karnofsky	Postop. Karnofsky	Resection	DEH	Surgical Approach
1	59/M	Epilepsy	C2–C4	Adenocarcinoma (prostate)	12	60%	80%	Total	4	Ant.
2	68/M	N/A	C2	Chordoma	14	50%	75%	Total	4	Post. */ Ant.
3	68/F	Art. hypertension	C2	Chordoma	15	55%	85%	Total	15	Post./ Ant.

DEH: days of hospital stay; Ant: anterior; Post: posterior. *: in other hospital.

## Data Availability

All of the data analyzed in this study are publicly available and on reasonable request from the corresponding author. Data available on request due to privacy.
